# Supplementary material for: Improving polygenic prediction with genetically inferred ancestry
Source: HGG Adv. 2022 Apr 20;3(3):100109. doi: 10.1016/j.xhgg.2022.100109 (PMC9095896; doi:10.1016/j.xhgg.2022.100109)
Supplement: Document S1. Tables S1 and S2 [file mmc1.pdf]

HGGA, Volume *3*

Supplemental information

Improving polygenic prediction with genetically inferred ancestry

Olivier Naret, Zoltan Kutalik, Flavia Hodel, Zhi Ming Xu, Pedro Marques-Vidal, and Jacques Fellay

Supplemental data

Table S1: Polygenic Score (PGS) details

	Target all			Target WBO					
	Base all						Base WBO		
**Phenotype**	**Thres**	**#SNPs**	R^2	**Thres**	**#SNPs**	R^2	**Thres**	**#SNPs**	R^2
Skin color	3.5e-4	34,127	0.0229	5e-5	4,235	0.0921	5e-5	2,151	0.0958
Menopause age	5e-5	1,050	0.0251	1e-4	1,296	0.0721	1e-4	1,133	0.0566
HBMD*	5e-5	5,002	0.0411	1.5e-4	5,404	0.1711	1e-4	4,209	0.1847
Blood pressure	2e-4	5,153	0.0231	0.003	20,508	0.0344	8.1e-3	37,888	0.0245
Menarche age	5e-5	3,247	0.0322	2e-4	4,258	0.0729	5e-5	1,914	0.0481
Baldness	5e-5	3,694	0.1210	5e-5	3,357	0.1246	1e-4	3,697	0.1347
BMI	2e-4	9,089	0.0751	0.0035	31,489	0.0861	4.5e-4	10,676	0.0742
Height	5e-5	14,745	0.2193	7e-4	26,629	0.2696	0.0018	35,561	0.2550
Educational	1.5e-4	2,739	0.0127	0.0051	28,096	0.0202	0.01	46,566	0.0167

Details of the PGS parameters produced by PRSice for the combination of base and target cohorts (base-UKB-all/target-UKB-all, base-UKB-all/target-UKB-WBO, or base-UKB-WBO/target-UKB-WBO). The p-value threshold for each PGS is determined by PRSice through cross-validation on the target-UKB cohort, limiting the PGS to a restricted number of SNPs. The R^2 reported correspond to the best-fit between the PGS and the phenotypes of the target-UKB cohort.

Table S2: Genome-wide association studies (GWAS) details

	Base all		Base WBO	
**Phenotype**	h^2_{GREML}	λ	h^2_{GREML}	λ
Skin color	0.221	1.184	0.284	1.216
Menopause age	0.228	1.130	0.224	1.127
HBMD*	0.409	1.372	0.410	1.385
Blood pressure	0.187	1.393	0.189	1.398
Menarche age	0.310	1.345	0.309	1.343
Baldness	0.440	1.280	0.456	1.292
BMI	0.301	1.682	0.305	1.727
Height	0.558	2.087	0.570	2.223
Educational	0.147	1.288	0.155	1.286

Details of the GWAS run with BOLT-LMM. The narrow-sense heritability is estimated with GCTA-GREML[1, 2]. The lambda indicates inflation. Elevated inflation has already been reported for a highly polygenic trait on UKB [3]

References

1. Yang J et al. Common SNPs explain a large proportion of the heritability for human height. Nature Genetics 2010 Jul; 42:565–9. DOI: 10.1038/ng.608. Available from: http://www.nature.com/doifinder/10.1038/ng.608 [Accessed on: 2016 Sep 26]
2. Yang J, Lee SH, Goddard ME, and Visscher PM. GCTA: A Tool for Genome-wide Complex Trait Analysis. American Journal of Human Genetics 2011 Jan; 88:76–82. DOI: 10.1016/j.ajhg.2010.11.011. Available from: http://www.ncbi.nlm.nih.gov/pmc/articles/PMC3014363/ [Accessed on: 2016 Nov 15]
3. Yengo L, Sidorenko J, Kemper KE, Zheng Z, Wood AR, Weedon MN, Frayling TM, Hirschhorn J, Yang J, and Visscher PM. Meta-analysis of genome-wide association studies for height and body mass index in ∼700000 individuals of European ancestry. en. Human Molecular Genetics 2018 Oct; 27:3641–9. DOI: 10.1093/hmg/ddy271. Available from: https://academic.oup.com/hmg/article/27/20/3641/5067845 [Accessed on: 2019 Feb 22]
