# Supplementary material for: Improving polygenic prediction with genetically inferred ancestry
Source: HGG Adv. 2022 Apr 20;3(3):100109. doi: 10.1016/j.xhgg.2022.100109 (PMC9095896; doi:10.1016/j.xhgg.2022.100109)
Supplement: Document S2. Article plus supplemental information [file mmc2.pdf]

HGG
Advances

ARTICLE

Improving polygenic prediction with genetically inferred ancestry

Olivier Naret,[1,2,*] Zoltan Kutalik,[2,3,6] Flavia Hodel,[1,2] Zhi Ming Xu,[1,2] Pedro Marques-Vidal,[5] and Jacques Fellay[1,2,4]

Summary

Genome-wide association studies (GWASs) have demonstrated that most common diseases have a strong genetic component from many genetic variants each with a small effect size. GWAS summary statistics have allowed the construction of polygenic scores (PGSs) estimating part of the individual risk for common diseases. Here, we propose to improve PGS-based risk estimation by incorporating genetic ancestry derived from genome-wide genotyping data. Our method involves three cohorts: a base (or discovery) for association studies, a target for phenotype/risk prediction, and a map for ancestry mapping; successively, (1) it generates for each individual in the base and target cohorts a set of principal components based on the map cohort—called mapped PCs, (2) it associates in the base cohort the phenotype with the mapped-PCs, and (3) it uses the mapped PCs in the target cohort to generate a phenotypic predictor called the ancestry score. We evaluated the ancestry score by comparing a predictive model using a PGS with one combining a PGS and an ancestry score. First, we performed simulations and found that the ancestry score has a greater impact on traits that correlate with ancestry-specific variants. Second, we showed, using UK Biobank data, that the ancestry score improves genetic prediction for our nine phenotypes to very different degrees. Third, we performed simulations and found that the more heterogeneous the base and target cohorts, the more beneficial the ancestry score is. Finally, we validated our approach under realistic conditions with UK Biobank as the base cohort and Swiss individuals from the CoLaus|PsyCoLaus study as the target cohort.

Introduction

Most common diseases of major public-health importance have a complex genetic architecture.[1–8] A polygenic score (PGS) (sometimes called polygenic risk score) is the weighted sum of risk alleles carried by an individual. By predicting a fraction of the risk of developing a disease, the PGS allows individuals to be stratified into different risk categories, with potential clinical value. For example, people who have a PGS in the upper 0.5% range have a 5-fold increased risk of developing coronary heart disease compared with the remainder of the population.[9] Such information could help reduce the risk of developing diseases by encouraging a healthier lifestyle or through preventive pharmacological interventions.[10] PGSs alone are already equal to or better than clinical risk models for predicting prostate cancer, breast cancer, and type 1 diabetes in the general population.[6,11,12] If their clinical utility is demonstrated, PGSs could be integrated into clinical practice in the coming years. Therefore, the practical limitations of their application must be urgently addressed.[13,14]

The phenotypic variance of a trait, V_P, is defined as $V_P = V_G + V_E$, with V_G representing the genetic variance, and V_E representing the environmental variance. In a multi-ancestry cohort, it is important to differentiate $V_{G,Individual}$, the fraction of V_G coming from variants shared between ancestries, and $V_{G,Ancestry}$, the fraction of V_G coming from variants that are ancestry specific. A fraction of V_E is also likely to be associated with ancestry $V_{E,ancestry}$. Thus, the phenotypic variance can be decomposed as follows:

$$V_P = \underbrace{V_{G,individual} + V_{G,ancestry}}_{Geneticfactor} + \underbrace{V_{E,ancestry} + V_{E,other}}_{Environmentalfactor}. \quad \text{(Equation 1)}$$

Genetic ancestry can be operationally defined as the systematic difference in allelic frequencies between subpopulations. It can be useful for biomedical applications[15] and is preferred to the concept of ethnicity.[16] For example, the current best integrative-risk model for coronary heart disease, “QRISK2,” includes a “self-reported ethnicity” risk parameter. Replacing it with genetic ancestry would (1) make the medical investigation more reliable by transforming it into a measurable biological variable detached from the notion of ethnicity, (2) improve the quality of the risk parameter by moving from a categorical to a continuous measure, and (3) allow the inclusion of individuals who do not know their ancestry or whose ancestry composition is uncertain.

Genetically, ancestry can be estimated via principal-component analysis (PCA) of genome-wide genotyping data to obtain the genetically inferred ancestry. The PCA produces a series of ordered axes, the first ones of which empirically correspond to the genetic ancestry.[17] By

[1]School of Life Sciences, École Polytechnique Fédérale de Lausanne, Lausanne, Switzerland; [2]Swiss Institute of Bioinformatics, Lausanne, Switzerland; [3]University Center for Primary Care and Public Health, Lausanne, Switzerland; [4]Precision Medicine Unit, Lausanne University Hospital and University of Lausanne, Lausanne, Switzerland; [5]Department of Medicine, Internal Medicine, Lausanne University Hospital and University of Lausanne, Lausanne, Switzerland; [6]Department of Computational Biology, University of Lausanne, Lausanne, Switzerland
*Correspondence: onaret@gmail.com
https://doi.org/10.1016/j.xhgg.2022.100109.
© 2022 The Authors. This is an open access article under the CC BY license (http://creativecommons.org/licenses/by/4.0/).

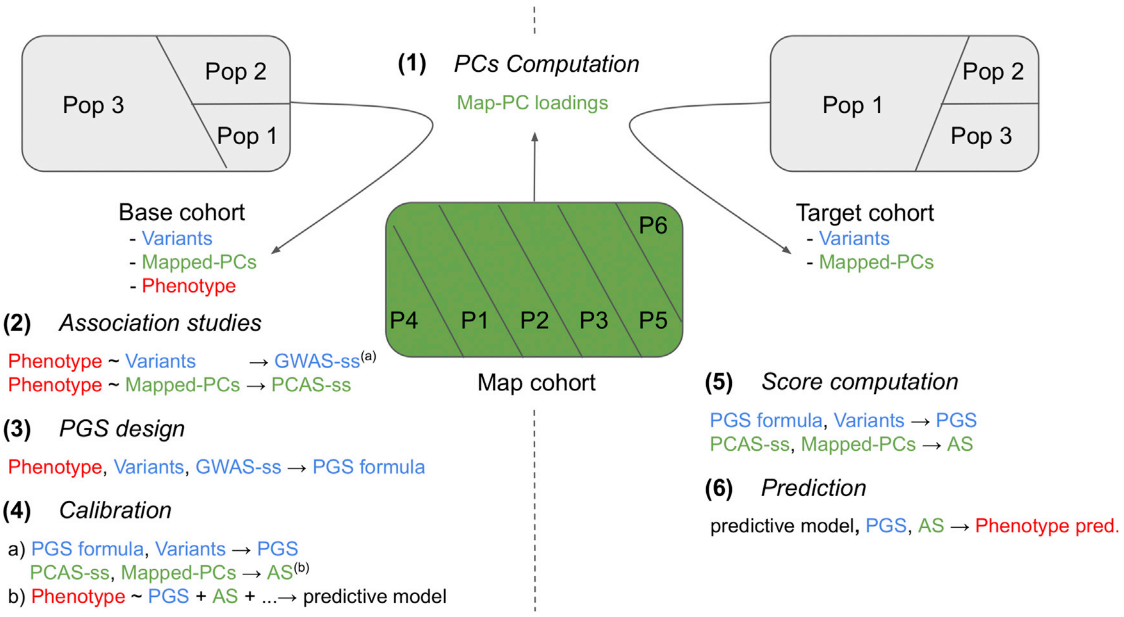

Figure 1. Overview of the method
Diagram of the components and steps of the method. The cohort map is used to define the PC space for the base and target cohorts. Summary statistics from the two types of association studies conducted in the base cohort (GWAS-ss and PCAS-ss) are used to calculate the PGS and AS on the target cohort. Finally, these two scores are used jointly as parameters of the predictive model. (A) Summary statistics. (B) Ancestry score.

definition, because PCA is an unsupervised machine-learning method, it does not require labels, which avoids confusion with ethnicity. Because it produces continuous variables, it allows a precise definition of the different components of an individual's ancestry.

In genome-wide association studies (GWASs), such genetically inferred ancestry can be used to correct for population stratification. Specifically, the coordinates of the relevant principal-component axes can be included as covariates in the association models together with other demographic or clinical risk parameters, such as age or sex. Subpopulation-specific variants will be responsible for the $V_{G,Ancestry}$ component and will covary strongly with the covariate carrying ancestry. Therefore, the PGS calculated from the resulting GWAS summary statistics only accounts for the $V_{G,Individual}$ component.

We here propose a method that improves PGS-based prediction of complex diseases using additional information about genetic ancestry derived from PCA. Through both simulation and real-life testing in large cohorts, we show that the addition of an ancestry score (AS) based on principal components, which takes into account the $V_{G,ancestry}$ component of the phenotypic variance, improves the results of predictive models.

Materials and methods

Workflow for improving phenotypic prediction with genetic ancestry

Association studies, such as a GWASs, are performed in what we call a base cohort. The "individual risk scores" or "phenotypic predictions" are calculated on what we call a target cohort. When we run a PCA on the base cohort, the populations separated with each principal component (PC) is cohort specific. Therefore, if in the base cohort we run an associations study between a set of PCs and a phenotype, it is necessary to have a similar set of PC variables in the target cohort if we want to use it for phenotypic predictions. Unfortunately, the genetic information of the base cohort is often not accessible due to privacy issues, and thus direct transformation between the two PC spaces can be impractical. Here, we propose a method to circumvent this problem with an intermediate map cohort that must be accessible to all stakeholders and have sufficient diversity to separate the populations present in the base and target cohorts.

The different steps of the method can be visualized in Figure 1. The separation between the two investigating entities is represented by the dotted line, only the map-cohort in the middle is accessible to both. We describe the generic workflow in the following section and specify in parentheses the specific tools and parameters we decided to use.

(1) Mapping the base cohort to the map cohort. First, the base cohort must be mapped to the PC space of the map cohort. To do this, the PC space of the map cohort is based on an optimal set of SNPs selected with respect to the base- and map-cohorts such that (1) only SNPs present in both are retained; (2) on the base-cohort side, a first filtering of SNPs to be kept in the analysis can be done, but we suggest not to discriminate on the basis of minor allele frequency (MAF) and linkage disequilibrium (LD) at this level (we filtered SNPs for imputation quality with INFO >0.9); on the map-cohort side, SNPs are filtered for MAF (we use MAF >0.01) and pruned (we use an LD threshold based on an R coefficient of $r2 = 0.5$ and a maximum base pair in the sliding window of $kb = 250$);[18] (3) the intersection

of these two subsets produces a set $S1$ of $s1$ reference SNPs. In the following section, we will refer in the mathematical notations to the base, target, map, and calibration cohorts with superscript b/t/m/c. A PCA is run on G^m, the genotype data matrix of the map cohort with $s1$ columns corresponding to the SNP subset S1, to produce the *map-PC SNP-loadings L*, a p times $s1$ matrix with p representing the number of PCs retained (we decided to retain 40 PCs). These *map-PC SNP-loadings* are used to produce set of PCs that we call mapped PCs for the base cohort such that

$$P^b = G^b \times L^T, \qquad \text{(Equation 2)}$$

where P^b is the matrix of size m_b times p and G^b here is the corresponding subset S1 of reference SNPs from the genotype data matrix of size m_b times $s1$.

(2) Associations analyses on the base cohort. Second, we perform two association studies for the phenotype of interest on the base cohort: a GWAS (Equation 3) and what we call a principal-component association study (PCAS; as Equation 4).

Let GWAS estimating the β_i^b effect size for each SNP i on the phenotype be a linear model such that

$$Y^b \sim \beta_i^b G_i^b + \gamma C^b, \qquad \text{(Equation 3)}$$

where within the base cohort, Y^b is the vector of phenotypes, G^b is the full genotype data matrix of size m_b times s, G_i^b is the column vector for the variant i, β_i^b is the corresponding effect size, C^b is the matrix of covariates of size m_b times c, with c as the number of covariates, and γ is the corresponding column vector of effect sizes. The outcome of the GWAS is the "GWAS summary statistics."

In our case, after quality control for imputation and MAFs (such that MAF $>1e^{-4}$ and INFO >0.8), we run the GWAS with BOLT-LMM.[19] It is worth noting that the GWAS method implemented in BOLT-LMM corrects for the population structure by using a mixed-effect model.[19]

For the PCAS, we fitted a multiple linear regression model to estimate for each mapped PC i in p the effect size b_i^b on the phenotype. Specifically, the model takes the form

$$Y^b \sim b_1^b P_1^b + b_2^b P_2^b + \ldots + b_p^b P_p^b + \gamma C^b, \qquad \text{(Equation 4)}$$

where $P_{b,i}$ is the column vector for the mapped PC i of size m_b, and b_i^b is the corresponding effect size.

For convenience, we call the resulting trained model "PCAS summary statistics."

In our case, for both association studies, the covariate C includes age at recruitment, sex, and genotyping array. In the GWAS model, as many as the 40 PCs computed by the UK Biobank are used to adjust for population structure. We chose to include 40 PCs, as it was shown in earlier studies that a deep subpopulation structure was found in UKB.[20]

(3) Defining PGS design. Third, to design the best PGS formula, a calibration cohort is needed to determine the SNPs to include in the PGS formula. Practically, it would be a small hold-out subset of the base cohort. We define the calibration cohort with the genotype data G^c, mapped PC P^c, and phenotype Y^c.

In our case, the PGS formula is estimated with PRSice on the calibration cohort by a strategy based on variant clumping and p value thresholding to determine the optimal set of SNP $S2$ to construct the PGS.[21] In short, the clumping step takes advantage of the LD properties of the genome to construct groups of SNPs (or clusters) below a given maximum p value threshold. These LD properties must either be computed from the target cohort or drawn from an external reference panel. We chose to use 1KG as the reference panel, with a maximum window for each clump of 250 kb and an r^2 cutoff of 0.01. From each clump, one SNP is selected for inclusion in the final set used to construct the PGS. PRSice then determines the optimal p value threshold used to retain the set of SNPs to calculate the PGS. The summary of the PGS is described in Table S2.

(4) Calibration of the predictive model. Fourth, the calculation of phenotypic predictions involves a calibrated predictive model in which the coefficients of different parameters such as age, sexm and other relevant covariates, but also here the PGS (β_{PGS}) and the AS (β_{AS}), have been estimated. To calibrate the predictive model, we use for a second time the calibration cohort.

The PGS is computed, such that for individual j

$$PGS_j^c = \sum_i^{s2} \widehat{\beta}_i^b \times G_{i,j}^c, \qquad \text{(Equation 5)}$$

where $\widehat{\beta}^b$ is the vector of the estimated SNP effect sizes and where G^c is the m_c times $s2$ matrix of genotype values.

The AS is computed, such that for individual j

$$AS_j^c = \sum_i^{p} \widehat{b}_i^b \times P_{i,j}^c, \qquad \text{(Equation 6)}$$

where $\widehat{b^b}$ is the vector of the estimated mapped-PC effect sizes and where P^c is the m_c times p matrix of mapped-PC values.

Finally, we calibrate our predictive model, a multiple linear regression model

$$Y^c \sim \beta_{PGS}^c PGS^c + \beta_{AS}^c AS^c + \gamma C^c, \qquad \text{(Equation 7)}$$

where $\beta_{PGS} PGS^c$ and $\beta_{AS} AS^c$ are the PGS and AS predictors, respectively, with their corresponding effect sizes that we want to estimate.

(5) Computation of ASs and PGSs on the target. Fifth, similarly, PGS^t and AS^t are computed for each of the m_t target samples with Equations 5 and 4 with P^t and G^t, the matrix of size m_t times p, and m_t times $s2$, respectively.

But for AS to get the mapped PC of the target cohort, Equation 2 is used beforehand on G^t to produce P^t. In that case, it is likely that some SNPs will be missing. A strategy based on the LD properties of the map cohort can be used to replace the missing SNPs with the best tagging SNPs (we take the SNP with the maximum $INFO \times r^2$).

(6) Phenotypic predictions. Finally, the calibrated predictive model can be used to calculate the phenotypic predictions

on the target samples, such that the phenotypic prediction for individual j is

$$\widehat{Y_j^t} = \widehat{\beta}_{PGS}^c \times PGS_j^t + \widehat{\beta}_{AS}^c \times AS_j^t + + \widehat{\gamma} C^t. \qquad \text{(Equation 8)}$$

Evaluation of AS

PGSs and ASs are two composite variables derived from the same genome-wide genotyping data. Therefore, in order to estimate the predictive value of the AS, it is necessary to evaluate it together with the PGS. Two predictive models are calibrated, one with PGS alone and the other with both the PGS and AS (Equation 7). The two predictive models are used on the target cohort to each generate a set of phenotypic predictions, $\widehat{Y}_{PGS}$ and $\widehat{Y}_{PGS+AS}$ (Equation 8). The resulting phenotypic-variance explained (PVE) is calculated by taking the R^2 regression score (coefficient of determination) between the prediction and the actual phenotypes.

Simulations

We generated genotyping and phenotypic data for a base cohort, a calibration cohort, and a target cohort, each based on a mixture of individuals from three genetically equidistant populations A, B, and C. The SNPs present in the simulated dataset can be non-specific (evenly distributed in the three populations), population specific (exclusive to one population), or stratified (present at different frequencies between populations). The alternative allele frequency for each population $A_F^{P_A}$, $A_F^{P_B}$, and $A_F^{P_C}$ was generated for each SNP with respect to its category. For population-specific SNPs, an allelic frequency is, with equal probability, either drawn from a uniform distribution $R_f \sim U(0.4, 1)$ or set to 1. The other two populations are attributed for the corresponding allele a frequency of 0. For stratified SNPs, we followed the model of Balding and Nichols[22] where a reference allele frequency (R_f) is first drawn from a uniform distribution $R_f \sim U(0, 1)$ and is used to derive the alternate allele frequencies from a β distribution for each population according to their F_{st} [23,24] such that

$$A_f \sim Beta\left(R_f \frac{(1\text{-}F_{st})}{F_{st}}, (1\text{-}R_f)\frac{(1\text{-}F_{st})}{F_{st}}\right),$$

with F_{st} varying between 0.02 for two populations and 0.2 for the third. Finally, for a non-specific SNP, an allelic frequency is drawn from a uniform distribution $U(0, 1)$ and is used by all samples.

Genotype data were generated based on A_f such that G, a matrix of size m times s with m as the number of samples, and s, the number of SNPs, respectively, with genotypes 0 (homozygous for the reference allele), 1 (heterozygous), or 2 (homozygous for the alternative allele) were assigned for a given population P with probabilities $(1\text{-}A_f^P)^2$, $2A_f^P(1\text{-}A_f^P)$, and $(A_f^P)^2$ respectively.

Phenotypes Y were generated based on heritability $h^2 = 0.6$ by adding two vector components of size m, the genetic basis Y_g and the environmental basis Y_e, such that, $Y = Y_g + Y_e$. Firstly, Y_g was generated from a total of s SNPs selected randomly as 10% of SNPs per category and associated with an effect size β_i^G drawn from a Gaussian distribution $\beta_i \sim \mathcal{N}(\mu = 0, \sigma^2 = 1)$, such that $Y_g = G \times \beta^G$ with β^G as the vector of the effect size. Secondly, based on the generated genetic component, μ_{Y_g} and $\sigma^2_{Y_g}$, its corresponding mean and variance, are calculated to generate Y_e. Finally, Y_e was drawn from a normal distribution such as $Y_e \sim \mathcal{N}\left(0, \sigma^2_{Y_g}\frac{1\text{-}h^2}{h^2}\right)$.

We computed a PCA on the base-cohort-generated genotyping data (G^b) to produce the corresponding PC P^b and PC loadings that we use to project the calibration and target cohorts into the same PC space and produce the mapped PCs $P^{c/t}$. We performed a GWAS as given by Equation 3 with PC1 and PC2 as covariates C and $\widehat{\beta}_i^G$ as the estimate of the SNP i effect size. On the calibration cohort, we determined the best set of SNP S' to build the PGS with a simple implementation of the p value thresholding method of PRSice. Based on this set of SNP S', we constructed the PGS of the calibration and target cohorts as given by Equation 5.

We performed a PCAS on the base cohort for the first two PCs—sufficient to differentiate 3 populations—following Equation 4 to estimate the effect sizes of the mapped PC $\widehat{\beta}^P$. We calculated the AS based on the top 2 associated PCs in the calibration and target cohorts following Equation 6.

Finally, we used the data from the calibration cohort to calibrate the risk model, following Equation 7. With the calibrated risk model, we performed a phenotypic prediction on the target cohort, so that the prediction for a sample j is given by Equation 8.

The code to reproduce the simulations is available on Github (see Web resources).

Cohorts

Map cohort: 1000 Genome Project

We used 1000 Genome Phase 3 dataset (1KG) publicly available online.[25,26] It contains 2,404 ethnically diverse samples classified into 5 superpopulations: European (EUR, n = 503), African (AFR, n = 661), Admixed America (AMR, n = 347), East Asian (EAS, n = 504), and South Asian (SAS, n = 489).

Base and target cohort: UK Biobank

We constructed base and target cohorts from the UK Biobank (UKB) based on all individuals (488,000) or white Britons only (407,000). The recruitment process was described previously.[27] Briefly, participants visited one of the UKB assessment centers between 2006 and 2010. The age range of participants at recruitment was 40–69 years (mean age 56.5 years, 8.1 years), with 54.2% female.

Genotyping and imputation of participants in the UKB study were fully described by Bycroft et al.[28,29] Briefly, samples were genotyped using the UK BiLEVE Axiom array (Affymetrix) (10.2%) or the UKB Axiom array (Applied Biosystems). Genotypes were phased using SHAPEIT3 with the 1KG phase 3 dataset as a reference and then imputed using the Haplotype Reference Consortium, 1KG phase 3, and UK10K data as reference panels. Participants were removed if their genetic sex did not match their reported sex, if they had a non-XX/XY sex-chromosome karyotype, or if they had excessive (>5%) missing genotyping.

Phenotypes were selected based on their high degree of differentiation between populations as characterized by the Global Distribution of Genetic Traits (GADGET).[30] Where necessary, phenotypes were normalized by rank-based inverse normal transformation and/or residualization by sex. The categorical phenotypes were turned into discrete variables. The details of the phenotypes are given in the Table 1. Summary statistics for the set of GWASs are available in the supplementary materials in Table S1.

External target cohort: CoLaus|PsyCoLaus

As external target cohort, we used the Cohorte lausannoise (CoLaus|PsyCoLaus), a population-based research study launched in 2003 in Lausanne, Switzerland, as an additional independent target cohort. It includes a total of 4,781 unrelated individuals of European ancestry after filtering out participants whose genetic sex did not match the reported sex or whose missing genotype rate was excessive (>5%). Participants ranged in age from 35 to 75 years at enrollment (mean ± SD: 51.1 ± 10.9), with 52.5% being female.[31]

Table 1. Phenotype details

Phenotype	F_{STAT}	Type	Transformation	Sample size	White only
Skin color	774	Cat(6)	Cont	478,929	403,189
Menopause age	499	Cont	INV	149,435	127,370
HBMD*	404	Cont	INV/Sex Res	274,000	237,166
Diastolic blood pressure	319	Cont	INV/Sex Res	455,457	381,383
Menarche age	172	Cont	INV	255,616	149,435
Baldness	162	Cat(4)	Cont	220,192	186,127
BMI	64	Cont	INV/Sex Res	484,587	406,956
Height	55	Cont	INV/Sex Res	485,043	407,318
Educational attainment	NA	Cont	INV	418,573	350,305

Distribution of the different phenotypes including the samples size, the ancestry (all samples versus White only), the type (continuous or categorical), the transformation procedure (INV, inverse normal transformation; Sex Res, residualised on sex; Cont, transformed from categorical phenotype to continuous), and FSTAT (degree of the phenotype difference between super populations). *Heel bone mineral density.

Genotype imputation was performed using two independent reference panels: the HRC reference panel and the merged 1000 Genomes phase 3 and UK10K reference panel.[32–34] Phasing and imputation were performed on the Sanger imputation service (https://imputation.sanger.ac.uk).

We used standing height, body mass index (BMI), and diastolic blood pressure as phenotypic outcomes. Phenotypes were normalized on the basis of the parameters used in the UKB phenotype normalization.

Results

Mapped-PC characterization

As a first step, we mapped individuals from both the base and target cohorts to the PC space of the map cohort. We characterized the resulting mapped PCs by assessing the correspondence between the position of the mapped PCs from UKB or CoLaus|PsyCoLaus and the PCs from the 1KG samples and, second, by testing whether the mapped PCs and the "regular" PCs explain the phenotypic variance with similar magnitude.

Visualization of the mapped PCs

We first jointly plot the mapped PCs of UKB/CoLaus|PsyCoLaus and the corresponding PCs of the map cohort (1KG). In Figure 2A, PC1 and PC2 from *UKB-all* and 1KG overlap widely, showing the diversity present in both cohorts. Figure 2B shows the PC5 and PC7 for *UKB-WBO* and the European 1KG samples. PC5 and PC7 are the axes discriminating the most samples of different European ancestry. As expected, there is a significant overlap of *UKB-WBO* with the British cluster (Great Britain [GBR]). Similar to CoLaus|PsyCoLaus, in Figure 2C, PC1 and PC2 validate that CoLaus|PsyCoLaus is exclusively composed of individuals of European ancestry. Specifically, in Figure 2D, PC5 and PC7 show that CoLaus|PsyCoLaus is broadly consistent with a Central European population, as expected for a Swiss cohort.

PVE by mapped PCs versus PCs

We then compared the PVE by the mapped PCs and by the regular PCs, which comes from a PCA done directly on the cohort (in CoLaus|PsyCoLaus) or provided (in UKB).[28,35] The regular PCs derived directly from the cohort can be considered as the upper bound when predicting a trait. We estimate the PVE by a multiple linear model based on the top 40 PCs of UKB (*target-UKB-all*) and CoLaus|PsyCoLaus with a 10-fold cross-validation. The results in Figures 3A and 3B show similar levels of PVE by the two approaches for the different phenotypes.

Evaluation of the AS

We evaluated the performance of the AS, the composite variable we created from mapped PCs that captures the association between phenotype and ancestry.

Simulations

For different trans-*ancestry genetic architectures*

To characterize scenarios where the use of ASs would lead to a gain in predictive power, we simulated the scenarios based on the different genetic architectures as shown in Figure 4A. The equilateral triangle represents a map with one of the populations A, B, or C at each vertex. The circles represent the number of SNPs per category, which can be non-specific (green in the middle, evenly distributed), population specific (red on the vertices, population exclusive), or stratified (blue in between). For all scenarios, the total number of SNPs is kept at a constant total of 500. Based on a scenario, data are generated for three sample sets—the base, calibration, and target cohorts—with 20,000, 7,000, and 3,000 samples from populations A, B, and C, respectively, for a total sample size of 30,000. We repeat the simulations 50 times per scenario.

The results of the different scenarios are shown in Figure 4B. We see in Figure 4B that adding the AS increases the PVE and decreases the mean square error (MSE) exclusively when there are population-specific causal variants (scenarios 1 and 3) but not when the causal SNPs are just stratified (scenario 2). This is due to the fact that because population-specific SNPs are co-linear to PCs, the

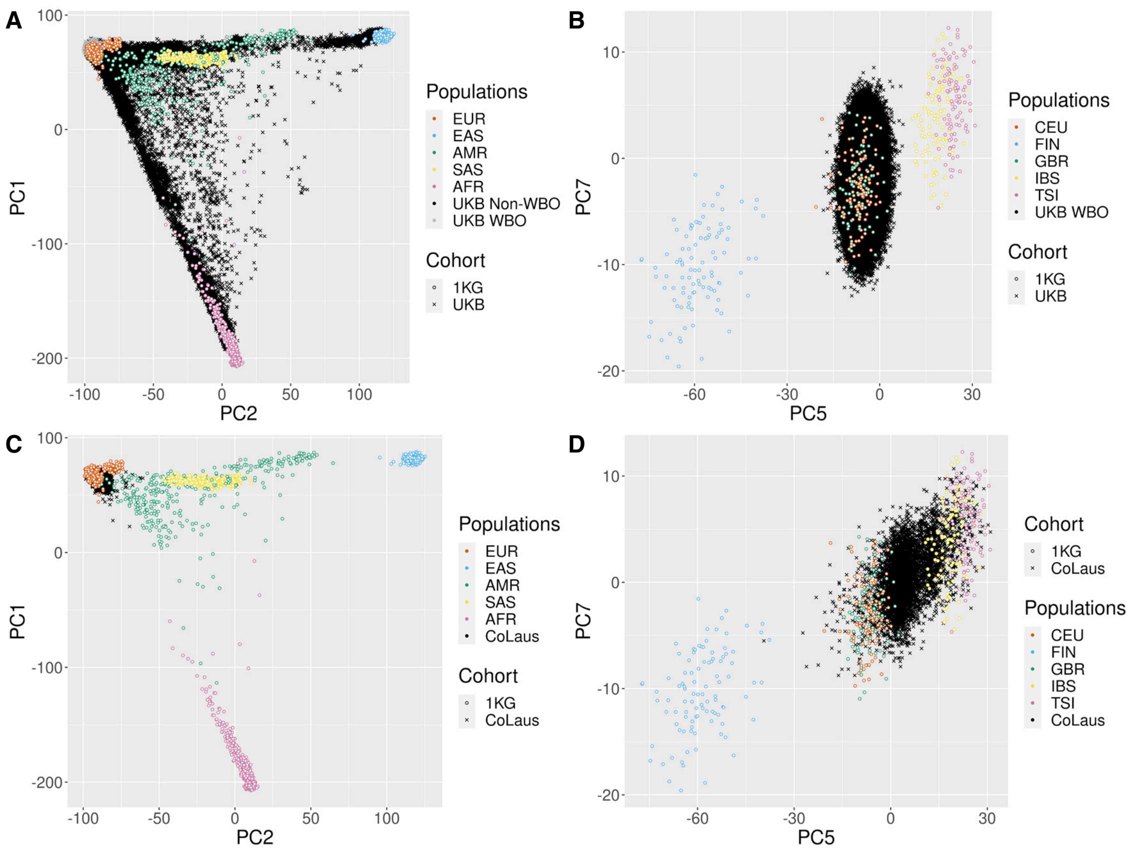

Figure 2. Projection of the cohorts in the 1KG PC space
(A) Projection of UKB: PC1-PC2.
(B) Projection of UKB: PC5-PC7.
(C) Projection of the CoLaus|PsyCoLaus: PC1–PC2.
(D) Projection of the CoLaus|PsyCoLaus: PC5–PC7.
PC plots of the UKB and CoLaus|PsyCoLaus mapped PCs (projected) with 1KG map PCs (projector). (A) shows the PC1 and PC2 of the entire UKB cohort with the 1KG cohort. (B) shows the PC5 and PC7 of the UKB-WBO cohort with the 1KG samples of European ancestry. (C) shows the PC1 and PC2 from CoLaus|PsyCoLaus with the 1KG cohort. (D) shows the PC5 and PC7 from CoLaus|PsyCoLaus with the 1KG samples of European ancestry only.

estimated SNP effect size is nullified. In contrast, causal stratified SNPs have their effect size corrected but are not discarded and end up included in the PGS. We conclude that, in real-world data, the increase in PVS due to the AS use could arise, along with covariant environmental factors, from similar population-specific SNPs.

For generalization. To evaluate the transferability of AS, we simulated scenarios based on the different cohort composition as shown in Figure 5A. We run simulations with different population structures for, on the one hand, the base and calibration cohorts and, on the other hand, the target cohort. The simulated genetic data correspond to scenario 3 of the previous simulation section (Figure 4A).

The results of the different scenarios are presented in Figure 5B. We generated scenarios 1 to 5 with increasing heterogeneity between the base/calibration cohorts and the target cohort. From scenarios 1 to 3, we go from a homogeneous situation in scenario 1 to proportions that are reversed in scenario 3. In all three scenarios, the models with PGSs alone see their mean PVE decrease and their variance as well as their MSE increase. Here, incorporating ASs to the model almost completely restores the mean EVP to its theoretical maximum of 0.6 and drastically reduces the variance. In scenario 4, the heterogeneity becomes extreme with a population C that corresponds to 3% of the base/calibration cohort and 97% of the target cohort. Here, the model with PGSs alone sees the variance of PVE explode while its mean continues to decrease. Even in this case, adding an AS to the model corrects for these effects strongly by decreasing the variance by a factor of 3 and bringing back the mean PVE above 0.4. In scenario 5, population A present in the base/calibration cohort is absent in the target cohort. In this case, the addition of an AS to the model almost completely corrects for cohort heterogeneity. Here, the model with only a PGS will estimate a shifted intercept on the calibration cohort relative to the optimal intercept for the target. This offset will be corrected by adding an AS to discriminate each population. We conclude that our method is effective to correct for cohort heterogeneity between the base/calibration cohorts and the target cohort.

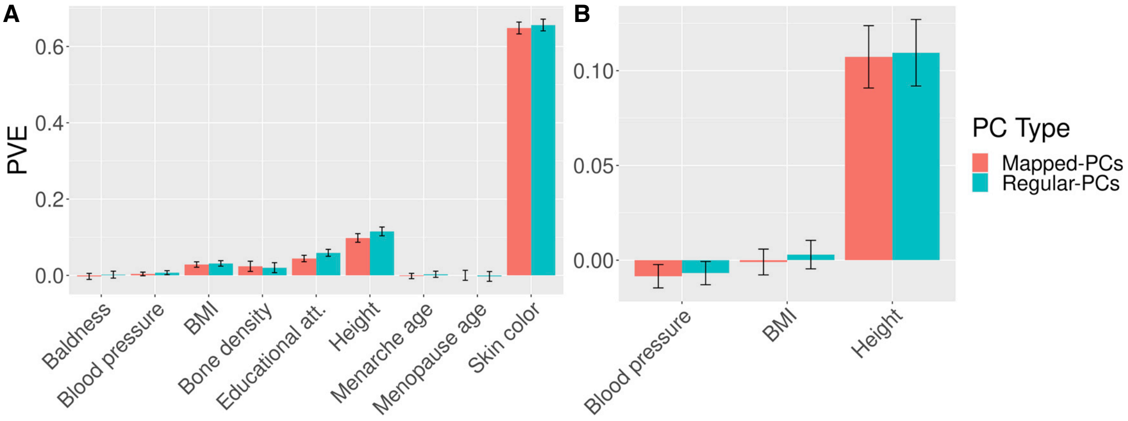

Figure 3. Comparison between mapped PCs and PCs of the association with phenotypes
(A) Within UK Biobank.
(B) Within CoLaus|PsyCoLaus.
Comparison of phenotypic variance explained between mapped PCs and PCs from UK Biobank (A) and CoLaus|PsyCoLaus (B). Error bars correspond to 95% of folds in the cross-validation process.

Application to real data

On UKB. We then evaluated our method on UKB. Two sets of base and target cohorts were generated: one with samples of White British ancestry only—*base/target-UKB-WBO*—and the other with samples of all ancestries—*base/target-UKB-all*. The split between the base and target cohorts was 90/10. The calibration cohort was generated multiple times based on a 10-times cross-validation from the target cohort.

Heritability is estimated by genome-based restricted maximum likelihood (GREML) method on the target cohort.[36,37]

The results based on *base/target-UKB-all* are shown in Figure 6A. The addition of the AS parameter increases the PVE for all phenotypes, with a variable magnitude. There is a small increase for diastolic blood pressure from 0.027 to 0.028, BMI from 0.075 to 0.079, and baldness from 0.120 to 0.128 and a larger increase for age of first menarche from 0.031 to 0.037, height from 0.219 to 0.271, and age at menopause from 0.024 to 0.039; it more than doubles for heel bone mineral density from 0.040 to 0.090 and education attainment from 0.012 to 0.054; and it is exacerbated for skin color from 0.023 to 0.654, where most of the PVE comes from the AS.

Results based on *base/target-UKB-WBO* in Figure 6B were used as a control to show that even when there is a population with narrow ancestry, the AS does not affect

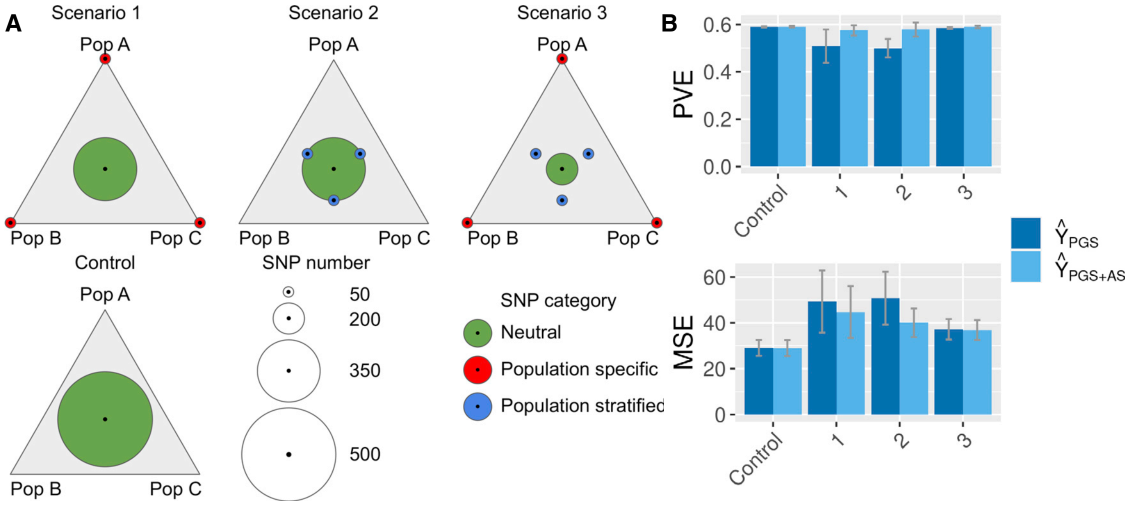

Figure 4. Simulations for different *trans*-ancestry genetic architectures
(A) Scenarios of *trans*-ancestry genetic architectures.
(B) Gain in phenotypic variance explained by the addition of the AS to the PGS.
(A) shows the different scenarios with either non-specific SNPs (scenario control), population-specific SNPs (scenario 1), stratified SNPs (scenario 2), or all SNPs (scenario 3). (B) shows the portion of PVE with PGS alone (light blue) or PGS combined with AS (dark blue), with bars corresponding to 95% of all simulations.

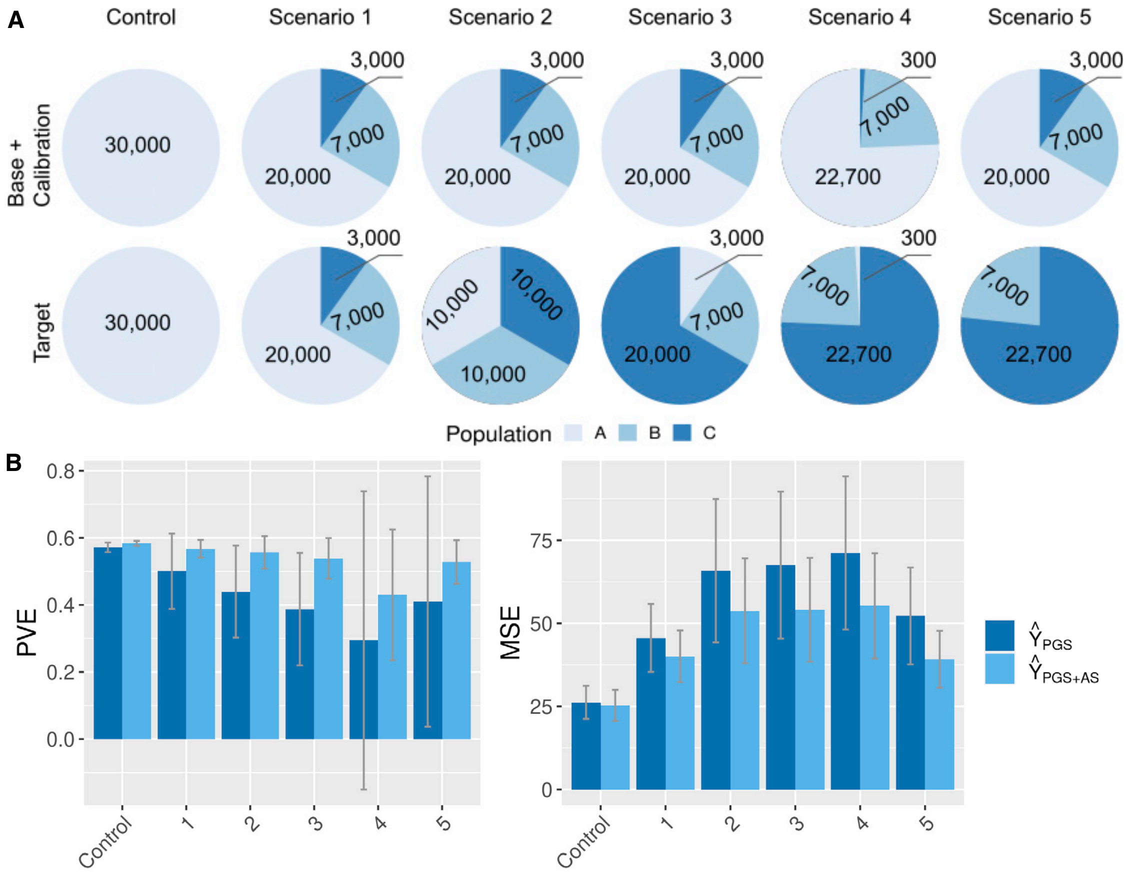

Figure 5. Simulations in case of cohort heterogeneity
(A) Cohort composition scenarios.
(B) Gain in phenotypic variance explained by the addition of AS to the PGS.
(A) shows the number of individuals within each population under different scenarios combining base and calibration cohorts on one side and target cohorts on the other side. The simulated genetic data correspond to scenario 3 of Figure 4A. (B) shows the portion of PVE with PGS only (light blue) or PGS combined with AS (dark blue), with bars corresponding to 95% of all simulations.

predictions. It was not significantly associated except for skin color, where it showed a slight gain.

Note that our exploration on the UKB data does not exploit the gain due to heterogeneity between cohorts as seen in the simulations (we are in a similar case to scenario 1).

On CoLaus|PsyCoLaus. We also evaluated our method using CoLaus|PsyCoLaus as an external target cohort. From UKB, two sets of base and calibration cohorts were generated, *base/calibration-UKB-WBO* and *base/calibration-UKB-all*, based on a 90/10 split.

Results based on *base/target-UKB-all* are shown in Figure 6C. For height, we observe a gain in PVE by adding the AS into the model of 15.6% (from 0.212 to 0.245). For BMI and diastolic blood pressure, as discussed in the previous sections (see Figures 3B and 6A), PCs and thus the AS do not explain the phenotypic variance. As a control, results based on *base/target-UKB-WBO* are shown in Figure 6B. As expected due to the homogeneous ancestry, we observe no gain or loss when adding the AS.

Note that with *base/target-UKB-WBO*, the PGS alone for height remains suboptimal, with a PVE of 0.223 compared with the model with the PGS and AS based on *base/target-UKB-all*.

These results show the generalizability of our method to an independent cohort. Of note, the best results were obtained by including all UKB individuals in the base cohort, even if the CoLaus|PsyCoLaus cohort consists exclusively of individuals of European ancestry.

Discussion

Here, we propose a method allowing for the inclusion of an ancestry parameter derived from genetic data into phenotype prediction scores without having to manually categorize individuals. We show that the inclusion of an AS improves prediction, especially for admixed populations. In addition, because our approach emphasizes the inclusion of all individuals, it tends to increase the statistical power and the production of summary statistics that generalize better to diverse populations. Our method could therefore promote a much-needed increase in population diversity for human genomic research.

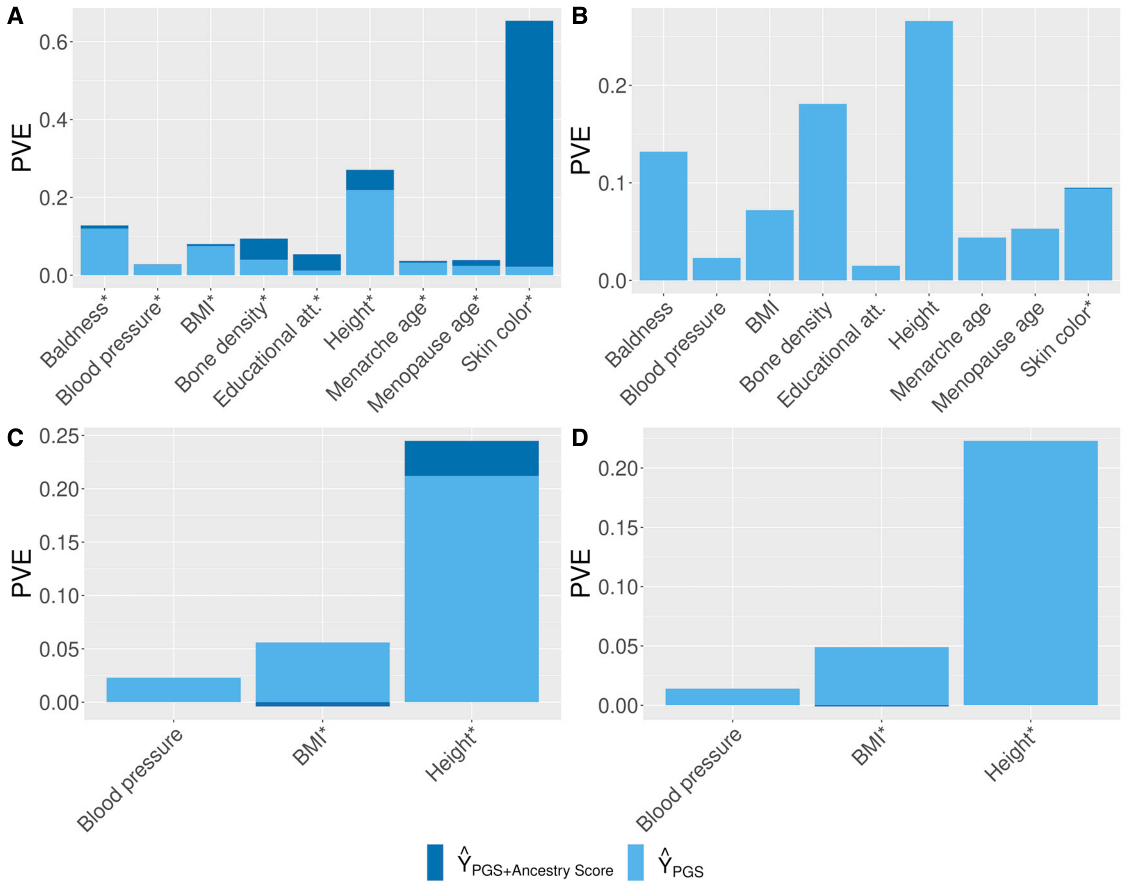

Figure 6. Evaluation with UK Biobank and CoLaus|PsyCoLaus
(A) Results on target-UKB-WBO with base-UKB-all.
(B) Results on target-UKB-WBO with base-UKB-WBO.
(C) Results on CoLaus|PsyCoLaus with base-UKB-all.
(D) Results on CoLaus|PsyCoLaus with base-UKB-WBO.
Portion of PVE with PGS only (light blue) or PGS combined with the AS (dark blue) for base and target UKB-all (A), UKB-WBO (B), or with base UKB-all and CoLaus|PsyCoLaus as a target (C) or UKB-WBO (D). *AS is significantly associated at calibration with $p < 0.05$.

So far, human genomic research has been disproportionately performed in populations of European ancestry, which might cause genomic-based medicine to exacerbate health disparities.[38] According to the GWAS catalog, although people of European descent make up only 16% of the world's population, they represent 79% of GWAS participants.[38,39] As a consequence, the predictive power of currently developed PGSs is lower in most underrepresented populations than in Europeans.[40] In addition, it has been reported that multi-ethnic GWASs or meta-GWASs increase statistical power for variants widely shared across populations,[41,42] which should lead to greater inclusion in future GWASs. As multi-ethnic GWASs become more common, the need to report the ancestry effect for the development of pan-ASs will increase.[43]

As a result of our simulations, we can hypothesize that the traits that show a higher gain in prediction upon inclusion of an AS are the ones that are influenced by more population-specific alleles with a phenotype that is also differentiated between populations. These population-specific alleles—fixed in one ancestry, absent in others—will only be detected as variables in a multi-ethnic GWAS and are perfectly correlated with ancestry. In such a context, they can be thought of as perfectly correlated variants that are distributed across the genome independently of their physical distances. In a GWAS, such variants are usually discarded because of their collinearity with a covariate controlling for ancestry. As a result, the corresponding GWAS summary statistics miss them entirely, which precludes their inclusion in downstream polygenic risk prediction. Here, we correct this bias by adding a separate ancestry term in the predictive risk model.

There are still limitations when doing predictions in a multi-ethnic setting. The PGS-based prediction will remain limited for *trans*-ethnic cases due to (1) variants in the target that are absent in the base GWAS, (2) different effect sizes for the same causal variant between two populations due to pleiotropic effect, and (3) unmatched tagging SNPs due to different LD structures between populations.

Non-genetic factors influence phenotypes in complex ways and must be carefully considered in genetic studies to avoid confounding and false genetic associations. In

particular, behavioral phenotypes are correlated with socio-economic and cultural factors, including racial and ethnic categories[44,45] that may be associated with ancestry. Consequently, the common GWAS assumption that environmental factors affect samples randomly does not hold, and the AS will be influenced by non-genetic factors. When studying such phenotypes, the investigator should not draw conclusions based solely on statistical associations between PCs and phenotype, as the socioeconomic factors that are captured could be misinterpreted as genetic or ancestry-related factors. Furthermore, because the magnitude and direction of associations between socioeconomic determinants and cultural background are society specific, the environmental effect embedded in the AS is less likely to be portable to a distant cohort.

Since genetic ancestry and ethnicity are closely related, it is necessary to draw the line between these two concepts. Ethnicity or race is a social construct that classifies people independently of the genetic component. Its meaning changes over time and between societies. Genetic ancestry—or genetically inferred ancestry—can be operationally defined as the systematic difference in allelic frequencies between subpopulations. Until now, reporting of ancestry has been mostly based on the Self-Identification of Race and Ethnicity (SIRE) method, which has important limitations,[46] such as its categorical rather than continuous nature; its overlap with the notion of race, whose definition fluctuates over time and depends on societies; it does not offer a simple solution for people of mixed ancestry, who are expected to become a larger share of the population in globalized societies;[47] and it does not allow for the classification of people who are unaware of their ancestry. To finely characterize genetic ancestry, we propose the use of mapped PCs, which can be easily derived from a reference map cohort such as the publicly available 1KG, to project any individual on a shared PC space. In addition to association studies, mapped PCs can also be shared to characterize ancestry in a discovery cohort as a new type of shareable metadata. Such data could, for example, be useful for assessing the compatibility between available GWAS summary statistics and a targeted individual for whom one wishes to calculate a PGS.

Here, we have shown that clinical risk models can benefit from a risk parameter, the AS, derived from mapped PCs, which allows each individual to be fitted to its phenotypic baseline value based on ancestry. The use of this fitting parameter makes it possible to directly apply the predictive models to individuals from underrepresented populations and of mixed ancestry.

The ClinGen Complex Disease Working Group has defined a standard method for reporting risk models based on PGSs[48] in collaboration with the Polygenic Score Catalog. The Polygenic Score Catalog is a rapidly growing repository for GWAS summary statistics.[49] This repository could host additional data useful for calculating risk parameters, such as PCAS summary statistics with the corresponding mapped-PC loadings. Today, researchers are strongly encouraged to share GWAS summary statistics to enable meta-analyses and speed up research. Similarly, we encourage researchers to share data to enable AS calculations.

We are at a pivotal moment for genomic-based medicine: large-scale personal data can begin to be used effectively to develop more individualized approaches to disease prevention and treatment. Ensuring equitable access to new approaches and technology is a major responsibility for the biomedical research community. We have introduced a method that aims to foster predictive models based on PGS and promotes the inclusion of more diverse populations in GWAS.

Data and code availability

The code that generated the simulated data during this study is the AS simulator and is available at Github (https://github.com/onaret/AS_simulator). The UKB and CoLaus|PsyCoLaus data supporting the current study have not been deposited in a public repository due to their sensitive nature.

Supplemental information

Supplemental information can be found online at https://doi.org/10.1016/j.xhgg.2022.100109.

Acknowledgments

We would like to express our gratitude to the members of the Pritchard lab for their guidance and thorough review of this work, especially Jonathan K. Pritchard, Roshni Patel, and Shaila Musharoff. This research was conducted via the UKB Resource under application number 27081. The CoLaus|PsyCoLaus study was and is supported by research grants from GlaxoSmithKline, the Faculty of Biology and Medicine of Lausanne, and the Swiss National Science Foundation (grants 3200B0–105993, 3200B0-118308, 33CSCO-122661, 33CS30-139468, 33CS30-148401, and 33CS30_177535/1). Data access: The CoLaus|PsyCoLaus cohort data used in this study cannot be fully shared as they contain potentially sensitive patient information. As discussed with the competent authority, the Research Ethic Committee of the Canton of Vaud, transferring or directly sharing this data would be a violation of the Swiss legislation aiming to protect the personal rights of participants. Non-identifiable, individual-level data are available for interested researchers who meet the criteria for access to confidential data sharing from the CoLaus Datacenter (CHUV, Lausanne, Switzerland). Instructions for gaining access to the CoLaus|PsyCoLaus data used in this study are available at https://www.colaus-psycolaus.ch/professionals/how-to-collaborate/

Received: November 10, 2021
Accepted: April 11, 2022

Web resources

AS simulator: https://github.com/onaret/AS_simulator.

References

1. Läll, K., Mägi, R., Morris, A., et al. (2017). Personalized risk prediction for type 2 diabetes: the potential of genetic risk scores. Genet. Med. *19*, 322–329. https://www.nature.com/articles/gim2016103.
2. Gatz, M., Reynolds, C.A., Fratiglioni, L., et al. (2006). Role of genes and environments for explaining alzheimer disease. Arch. Gen. Psychiat. *63*, 168–174. https://jamanetwork.com/journals/jamapsychiatry/fullarticle/209307.
3. Zheutlin, A.B., Dennis, J., Restrepo, N., et al. (2018). Penetrance and pleiotropy of polygenic risk scores for schizophrenia in 90,000 patients across three healthcare systems. Preprint at bioRxiv. https://www.biorxiv.org/content/early/2018/09/18/421164.
4. Howard, D.M., Adams, M.J., Clarke, T.K., et al. (2019). Genome-wide meta-analysis of depression identifies 102 independent variants and highlights the importance of the prefrontal brain regions. Nat. Neurosci. *22*, 343. https://www.nature.com/articles/s41593-018-0326-7.
5. Mavaddat, N., Michailidou, K., Dennis, J., et al. (2018). Polygenic risk scores for prediction of breast cancer and breast cancer subtypes. Am. J. Hum. Genet. *104*, 21–34.
6. Schumacher, F.R., Al Olama, A.A., Berndt, S.I., et al. (2018). Association analyses of more than 140,000 men identify 63 new prostate cancer susceptibility loci. Nat. Genet. *50*, 928–936.
7. Ruth, McPherson, and Anne, Tybjaerg-Hansen (2016). Genetics of coronary artery disease. Circ. Res. *118*, 564–578. https://www.ahajournals.org/doi/full/10.1161/CIRCRESAHA.115.306566.
8. Clarke, S.L., and Assimes, T.L. (2018). Genome-wide association studies of coronary artery disease: recent progress and challenges ahead. Curr. Atheroscler. Rep. *20*, 47. https://doi.org/10.1007/s11883-018-0748-4.
9. Khera, A.V., Chaffin, M., Aragam, K.G., et al. (2018). Genome-wide polygenic scores for common diseases identify individuals with risk equivalent to monogenic mutations. Nat. Genet. *50*, 1219–1224. http://www.nature.com/articles/s41588-018-0183-z.
10. Khera, A.V., Emdin, C.A., Drake, I., et al. (2016). Genetic risk, adherence to a healthy lifestyle, and coronary disease. N. Engl. J. Med. *375*, 2349–2358.
11. Sharp, S.A., Rich, S.S., Wood, A.R., et al. (2019). Development and standardization of an improved type 1 diabetes genetic risk score for use in newborn screening and incident diagnosis. Diabetes Care *42*, 200–207.
12. Maas, P., Barrdahl, M., Joshi, A.D., et al. (2016). Breast cancer risk from modifiable and nonmodifiable risk factors among white women in the United States. JAMA Oncol. *2*, 1295–1302.
13. Torkamani, A., Wineinger, N.E., and Topol, E.J. (2018). The personal and clinical utility of polygenic risk scores. Nat. Rev. Genet. *19*, 581–590. https://www.nature.com/articles/s41576-018-0018-x.
14. Lambert, S.A., Abraham, G., and Inouye, M. (2019). Towards clinical utility of polygenic risk scores. Hum. Mol. Genet. *28*, R133–R142. https://academic.oup.com/hmg/article/28/R2/R133/5540980.
15. Vyas, D.A., Eisenstein, L.G., and Jones, D.S. (2020). Hidden in plain sight – reconsidering the use of race correction in clinical algorithms. N. Engl. J. Med. *383*, 874–882.
16. Khan, A., McHugh, C., Conomos, M.P., et al. (2021). Guidelines on the Use and Reporting of Race, Ethnicity, and Ancestry in the NHLBI Trans-omics for Precision Medicine (TOPMed) Program (NHLBI Trans-Omics for Precision Medicine).
17. Price, A.L., Patterson, N.J., Plenge, R.M., et al. (2006). Principal components analysis corrects for stratification in genome-wide association studies. Nat. Genet. *38*, 904–909. http://www.nature.com/ng/journal/v38/n8/full/ng1847.html.
18. Purcell, S., Neale, B., Todd-Brown, K., et al. (2007). PLINK: a tool set for whole-genome association and population-based linkage analyses. Am. J. Hum. Genet. *81*, 559–575.
19. Loh, P.R., Tucker, G., Bulik-Sullivan, B.K., et al. (2015). Efficient Bayesian mixed-model analysis increases association power in large cohorts. Nat. Genet. *47*, 284–290. http://www.nature.com/doifinder/10.1038/ng.3190.
20. Canela-Xandri, O., Rawlik, K., and Tenesa, A. (2018). An atlas of genetic associations in UK Biobank. Nat. Genet. *50*, 1593–1599. https://www.nature.com/articles/s41588-018-0248-z.
21. Euesden, J., Lewis, C.M., and O'Reilly, P.F. (2015). PRSice: polygenic risk score software. Bioinformatics (Oxford, England) *31*, 1466–1468.
22. Balding, D.J., and Nichols, R.A. (1995). A method for quantifying differentiation between populations at multi-allelic loci and its implications for investigating identity and paternity. Genetica *96*, 3–12.
23. Holsinger, K.E., and Weir, B.S. (2009). Genetics in geographically structured populations: defining, estimating and interpreting F(ST). Nat. Rev. Genet. *10*, 639–650.
24. Wright, S. (1951). The genetical structure of populations. Ann. Eugenics *15*, 323–354.
25. Auton, A., Abecasis, G.R., Altshuler, D.M., et al. (2015). A global reference for human genetic variation. Nature *526*, 68–74. http://www.nature.com/doifinder/10.1038/nature15393.
26. Siva, N. (2008). 1000 Genomes project. Nat. Biotechnol. *26*, 256–266. https://www.nature.com/articles/nbt0308-256b.
27. Sudlow, C., Gallacher, J., Allen, N., et al. (2015). UK Biobank: an open access resource for identifying the causes of a wide range of complex diseases of middle and old age. PLoS Med. *12*, e1001779.
28. Bycroft, C., Freeman, C., Petkova, D., et al. (2018). The UK Biobank resource with deep phenotyping and genomic data. Nature *562*, 203. https://www.nature.com/articles/s41586-018-0579-z.
29. Bycroft, C., Freeman, C., Petkova, D., et al. (2017). Genome-wide genetic data on 500,000 UK Biobank participants. Preprint at bioRxiv. https://www.biorxiv.org/content/early/2017/07/20/166298.
30. Chande, A.T., Wang, L., Rishishwar, L., et al. (2018). GlobAl Distribution of GEnetic Traits (GADGET) web server: polygenic trait scores worldwide. Nucleic Acids Res. *46*, W121–W126. https://academic.oup.com/nar/article/46/W1/W121/4999244.
31. Firmann, M., Mayor, V., Vidal, P.M., et al. (2008). The CoLaus study: a population-based study to investigate the epidemiology and genetic determinants of cardiovascular risk factors and metabolic syndrome. BMC Cardiovasc. Disord. *8*, 6. https://doi.org/10.1186/1471-2261-8-6.
32. Loh, P.R., Danecek, P., Palamara, P.F., et al. (2016). Reference-based phasing using the Haplotype reference Consortium

panel. Nat. Genet. *48*, 1443–1448. http://www.nature.com/doifinder/10.1038/ng.3679.
33. Birney, E., and Soranzo, N. (2015 Oct). Human genomics: the end of the start for population sequencing. Nature *526*, 52–53.
34. Walter, K., Min, J.L., Huang, J., et al. (2015). The UK10K project identifies rare variants in health and disease. Nature *526*, 82–90. https://www.nature.com/articles/nature14962.
35. Abraham, G., and Inouye, M. (2014). Fast principal component analysis of large-scale genome-wide data. PLoS One *9*, e93766.
36. Yang, J., Benyamin, B., McEvoy, B.P., et al. (2010). Common SNPs explain a large proportion of the heritability for human height. Nat. Genet. *42*, 565–569. http://www.nature.com/doifinder/10.1038/ng.608.
37. Yang, J., Lee, S.H., Goddard, M.E., et al. (2011). GCTA: a tool for genome-wide complex trait analysis. Am. J. Hum. Genet. *88*, 76–82. http://www.ncbi.nlm.nih.gov/pmc/articles/PMC3014363/.
38. Martin, A.R., Kanai, M., Kamatani, Y., et al. (2019). Clinical use of current polygenic risk scores may exacerbate health disparities. Nat. Genet. *51*, 584. https://www.nature.com/articles/s41588-019-0379-x.
39. Petrovski, S., and Goldstein, D.B. (2016). Unequal representation of genetic variation across ancestry groups creates healthcare inequality in the application of precision medicine. Genome Biol. *17*, 157. https://doi.org/10.1186/s13059-016-1016-y.
40. Duncan, L., Shen, H., Gelaye, B., et al. (2019). Analysis of polygenic risk score usage and performance in diverse human populations. Nat. Commun. *10*, 1–9. https://www.nature.com/articles/s41467-019-11112-0.
41. Márquez-Luna, C., Loh, P.R., and Price, A.L. (2017). Multiethnic polygenic risk scores improve risk prediction in diverse populations. Genet. Epidemiol. *41*, 811–823. https://www.ncbi.nlm.nih.gov/pmc/articles/PMC5726434/.
42. Dikilitas, O., Schaid, D.J., Kosel, M.L., et al. (2020). Predictive utility of polygenic risk scores for coronary heart disease in three major racial and ethnic groups. Am. J. Hum. Genet. *106*, 707–716.
43. Eisenstein, M. (2021). Ranking the risk of heart disease. Nature *594*, S6–S7. https://www.nature.com/articles/d41586-021-01452-7.
44. Young, A.I. (2019). Solving the missing heritability problem. PLoS Genet. *15*, e1008222. https://journals.plos.org/plosgenetics/article?id=10.1371/journal.pgen.1008222.
45. Birney, E., Inouye, M., Raff, J., et al. (2021). The language of race, ethnicity, and ancestry in human genetic research. Preprint at arXiv. https://doi.org/10.48550/arXiv.2106.10041.
46. Morales, J., Welter, D., Bowler, E.H., et al. (2018). A Standardized Framework for Representation of Ancestry Data in Genomics Studies, with Application to the NHGRI-EBI GWAS Catalog, *19* (Genome Biology), pp. 1–10. https://www.ncbi.nlm.nih.gov/pmc/articles/PMC5815218/.
47. Sandra, L., and Colby, J.M.O. (2015). Projections of the Size and Composition of the U.S: 2014-2060 (The United States Census Bureau). Library Catalog: www.census.gov Section: Government. https://www.census.gov/library/publications/2015/demo/p25-1143.html.
48. Wand, H., Lambert, S.A., Tamburro, C., et al. (2020). Improving reporting standards for polygenic scores in risk prediction studies. Preprint at medRxiv. https://doi.org/10.1101/2020.04.23.20077099v1.
49. Lambert, S.A., Gil, L., Jupp, S., et al. (2020). The Polygenic Score Catalog: an open database for reproducibility and systematic evaluation. Preprint at medRxiv. https://doi.org/10.1101/2020.05.20.20108217v1.

HGGA, Volume *3*

Supplemental information

Improving polygenic prediction

with genetically inferred ancestry

Olivier Naret, Zoltan Kutalik, Flavia Hodel, Zhi Ming Xu, Pedro Marques-Vidal, and Jacques Fellay

Supplemental data

Table S1: Polygenic Score (PGS) details

	Target all			Target WBO					
	Base all						Base WBO		
**Phenotype**	**Thres**	**#SNPs**	R^2	**Thres**	**#SNPs**	R^2	**Thres**	**#SNPs**	R^2
Skin color	3.5e-4	34,127	0.0229	5e-5	4,235	0.0921	5e-5	2,151	0.0958
Menopause age	5e-5	1,050	0.0251	1e-4	1,296	0.0721	1e-4	1,133	0.0566
HBMD*	5e-5	5,002	0.0411	1.5e-4	5,404	0.1711	1e-4	4,209	0.1847
Blood pressure	2e-4	5,153	0.0231	0.003	20,508	0.0344	8.1e-3	37,888	0.0245
Menarche age	5e-5	3,247	0.0322	2e-4	4,258	0.0729	5e-5	1,914	0.0481
Baldness	5e-5	3,694	0.1210	5e-5	3,357	0.1246	1e-4	3,697	0.1347
BMI	2e-4	9,089	0.0751	0.0035	31,489	0.0861	4.5e-4	10,676	0.0742
Height	5e-5	14,745	0.2193	7e-4	26,629	0.2696	0.0018	35,561	0.2550
Educational	1.5e-4	2,739	0.0127	0.0051	28,096	0.0202	0.01	46,566	0.0167

Details of the PGS parameters produced by PRSice for the combination of base and target cohorts (base-UKB-all/target-UKB-all, base-UKB-all/target-UKB-WBO, or base-UKB-WBO/target-UKB-WBO). The p-value threshold for each PGS is determined by PRSice through cross-validation on the target-UKB cohort, limiting the PGS to a restricted number of SNPs. The R^2 reported correspond to the best-fit between the PGS and the phenotypes of the target-UKB cohort.

Table S2: Genome-wide association studies (GWAS) details

	Base all		Base WBO	
**Phenotype**	h^2_{GREML}	λ	h^2_{GREML}	λ
Skin color	0.221	1.184	0.284	1.216
Menopause age	0.228	1.130	0.224	1.127
HBMD*	0.409	1.372	0.410	1.385
Blood pressure	0.187	1.393	0.189	1.398
Menarche age	0.310	1.345	0.309	1.343
Baldness	0.440	1.280	0.456	1.292
BMI	0.301	1.682	0.305	1.727
Height	0.558	2.087	0.570	2.223
Educational	0.147	1.288	0.155	1.286

Details of the GWAS run with BOLT-LMM. The narrow-sense heritability is estimated with GCTA-GREML[1, 2]. The lambda indicates inflation. Elevated inflation has already been reported for a highly polygenic trait on UKB [3]

References

1. Yang J et al. Common SNPs explain a large proportion of the heritability for human height. Nature Genetics 2010 Jul; 42:565–9. DOI: 10.1038/ng.608. Available from: http://www.nature.com/doifinder/10.1038/ng.608 [Accessed on: 2016 Sep 26]
2. Yang J, Lee SH, Goddard ME, and Visscher PM. GCTA: A Tool for Genome-wide Complex Trait Analysis. American Journal of Human Genetics 2011 Jan; 88:76–82. DOI: 10.1016/j.ajhg.2010.11.011. Available from: http://www.ncbi.nlm.nih.gov/pmc/articles/PMC3014363/ [Accessed on: 2016 Nov 15]
3. Yengo L, Sidorenko J, Kemper KE, Zheng Z, Wood AR, Weedon MN, Frayling TM, Hirschhorn J, Yang J, and Visscher PM. Meta-analysis of genome-wide association studies for height and body mass index in ∼700000 individuals of European ancestry. en. Human Molecular Genetics 2018 Oct; 27:3641–9. DOI: 10.1093/hmg/ddy271. Available from: https://academic.oup.com/hmg/article/27/20/3641/5067845 [Accessed on: 2019 Feb 22]
